# Peer-reviewed publications in orthopaedic surgery from lower income countries: A comparative analysis

**DOI:** 10.1051/sicotj/2023039

**Published:** 2024-02-01

**Authors:** Sanjeev Sabharwal, Andrea Leung, Patricia Rodarte, Gurbinder Singh, Joel Johansen Bwemelo, Annelise S. Taylor, Josephine Tan, Richard Trott

**Affiliations:** 1 UCSF Benioff Children’s Hospital Oakland, Department of Orthopaedic Surgery 747 52nd Street Oakland CA 94609 USA; 2 Institute of Global Orthopaedics and Traumatology (IGOT) 2540 23rd Street, Building 7 San Francisco CA 94110 USA; 3 Muhimbili Orthopaedic Institute Kalenga Street West Upanga Dar es Salaam Tanzania

**Keywords:** Peer-reviewed publications, Low and middle income country, LMIC authors, Bibliometric study

## Abstract

*Introduction*: Musculoskeletal (MSK) disease is a substantial global burden, especially in lower income countries. However, limited research has been published on MSK health by scholars from these countries. We aimed to study the distribution of authorships, including trends in peer-reviewed orthopaedic publications based on each author’s affiliated institution’s country income status. *Methods*: Based on a bibliometric search, 119 orthopaedic-related journals were identified using the Journal Citation Reports database. Details of all scientific articles published in these journals between 2012 and 2021 were used to study trends and association between each of the author’s affiliated institution’s country income status, using the World Bank Classification. *Results*: Of the 133,718 unique articles, 87.6% had at least one author affiliation from a high-income country (HIC), 7.0% from an upper-middle income country (UMIC), 5.2% from a lower-middle income country (LMIC), and 0.2% from a low-income country (LIC). Overall, these articles were cited 1,825,365 times, with 92.5% of citations from HIC-affiliated authors and < 0.1% from LIC-affiliated authors. Over the 10-year study period, HIC-affiliated articles demonstrated the largest increase in the number of publications (9107–14,619), compared to UMIC-affiliated (495–1214), LMIC-affiliated (406–874), and LIC-affiliated articles (4–28). *Conclusions*: There are large and persistent disparities in orthopaedic research publications based on the country income status of the author’s affiliated institution, especially in the higher impact orthopaedic journals. Efforts should be made to increase opportunities for scholars from LICs and LMICs to publish their research in high-impact orthopaedic journals.

## Introduction

Musculoskeletal (MSK) disease is an enormous economic and societal burden worldwide and disproportionately impacts individuals in lower income countries [1, 2]. Furthermore, orthopaedic injuries make up the majority of non-fatal injuries due to increased road traffic accidents and less developed trauma care systems in the lower income countries [3, 4]. However, there remains limited research on musculoskeletal health done on this segment of the global population, especially by scholars from lower income countries [5].

Approximately 30% of studies published in medical journals on health issues in low-income countries (LICs) or lower-middle income countries (LMICs) lack any local authors [6, 7]. Orthopedic journals with higher impact factors are less likely to have a first or last author from an LIC/LMIC compared to journals with a lower impact factor [8]. While collaboration between investigators representing disparate populations can enhance the quality of research conducted in low-resource settings, the trends, potential impact and individual contributions from investigators from LICs/LMICs in orthopaedics remains largely unknown.

The purpose of our bibliometric study was to examine the trends, author affiliations, open access and post-publication citations of peer-review publications in orthopedic surgery based on the country income status of the authors’ affiliated institution.

## Materials and methods

In consultation with our institution’s library, we utilized the Journal Citation Reports (JCR) subscription database from Clarivate Analytics to identify journals listed in the “orthopedics” category as of the 2020 release [9]. This category contained 119 journals, including 82 titles in Science Citation Index Expanded (SCIE) and 37 titles in Emerging Sources Citation Index (ESCI). SCIE journals met Clarivate’s quality criteria for indexing in the Web of Science Core Collection along with their impact criteria by calculating a *Journal Impact Factor,* signaling their influence within their respective field [10]. ESCI journals met the quality criteria but not the impact criteria and therefore did not have a calculated impact factor in the 2020 release [11]. Additionally, we accessed Clarivate’s Web of Science (WoS) Expanded application programming interface (API) to export details of all articles published in the 119 orthopaedic journals over the last 10 years (from 2012 to 2021 inclusive) [12]. This API provides records from the WoS Core Collection, a bibliographic database including articles from over 21,000 scholarly journals which also tracks citations to these articles from other authors [13].

Results classified as “Article” (original research articles) and “Article; Early Access” were utilized for further analysis and all other document types were removed [14]. Included articles were limited to English language publications. There were 133,718 unique articles selected for the primary analysis. Non-research articles (such as Letter to the Editor and Review articles) and non-English language publications were excluded. Specifics of each of the remaining articles including the number of citations as tracked by WoS at the time of export, Web of Science Core Collection category (ESCI or SCIE), journal type, funding status, and Open Access Gold status was extracted. Open access gold (OAG) indicates the article was published for all to access freely on the journal web site and frequently incurs an author-based fee [15].

Country income was defined according to each country’s status in the World Bank Classification for the 2023 fiscal year [16]. The corresponding author’s affiliated institution’s country income level was identified. Articles with author’s affiliated institutions listed from countries representing two or more of the four income categories – low income (LIC), lower-middle income (LMIC), upper-middle income (UMIC), and high income (HIC) – were also identified and counted individually. This corresponded to the total number of 138,492 affiliations that were included for subgroup analysis.

### Data generation

The 119 journal titles as identified in the JCR Orthopedics category were first searched in the Publication Titles field in WoS to retrieve their nomenclature in the database (see Appendix A).

### Data analysis

Chi-square tests and Wald Odds ratios were used to assess the relationship between the listed author-affiliated institution’s country income status and categorical variables associated with each published article such as category of journal (SCIE vs. ESCI), acknowledgment of funding available for conducting the study and Open Access Gold status. An analysis of variance (ANOVA) test was conducted to evaluate the association of the number of citations of each article across the four income categories. A significance level of 0.05 (*p* < 0.05) was used to determine statistical significance. All statistical analyses were performed using R software, Version 1.2.5033.

## Results

### General characteristics and trends (Table 1)

**Figure 1:**
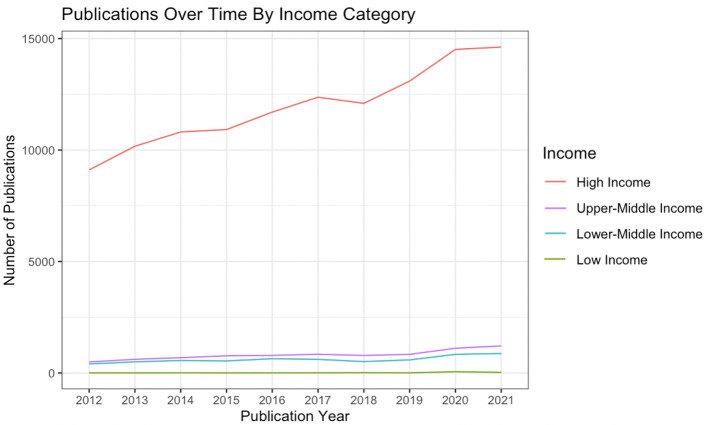
Number of orthopaedic publications over the study period (2012–2021) stratified by the corresponding author’s affiliated institution’s country income level, using the World Bank classification.

All 133,718 unique articles had author-institution affiliations listed with countries classified by the World Bank Classification for the current fiscal year. Of these publications, 87.6% had at least one author affiliation from a HIC, 7.0% from UMIC, 5.2% from LMIC, and 0.2% from LIC. Majority of the corresponding authors (87.5%) were from HICs and only 0.1% from LICs. Only 74 of the 133,718 articles had authors from both an HIC- and LIC-affiliated institutions, and 1,762 had HIC- and LMIC-affiliated authors. Between 2012 and 2021, there was a substantial increase in the total number of unique orthopedic articles from 10,012 in 2012 to 16,735 in 2021 (Figure 1). Stratifying by income category, HIC-affiliated authors had the largest absolute increase in number of published articles (9107–14,619), compared to UMIC-affiliated (495–1214), LMIC-affiliated (406 to 874), and LIC-affiliated (4–28) authors (Table 1).

**Table 1:** Bibliometric details of scientific articles published in 119 orthopaedic-related journals between 2012 and 2021, based on the author’s institutional affiliation using the World Bank Classification [16].

	High income (HIC) (%)	Upper middle income (UMIC) (%)	Lower middle income (LMIC) (%)	Low income (LIC) (%)	Total
Total articles with income data					133,718
Total number of affiliations	121,373 (87.6)	9677 (7.0)	7266 (5.2)	216 (0.2)	138,492
Corresponding authors	93,139 (87.5)	5593 (5.3)	7608 (7.1)	134 (0.1)	106,474
Number of citations	1,688,408 (92.5)	84,530 (4.6)	51,134 (2.8)	1293 (0.1)	1,825,365
Web of Science					
Emerging Sources Citation Index (ESCI)	12,422 (74.4)	1267 (7.6)	2927 (17.5)	91 (0.5)	16,707
Science Citation Index Expanded (SCIE)	102,118 (89.3)	8010 (7.0)	4143 (3.6)	106 (0.1)	114,365
NA	6833 (92.3)	400 (5.4)	156 (2.1)	19 (0.2)	7,408
Funding					
Yes	42,355 (92.2)	2251 (4.9)	1224 (2.7)	95 (0.2)	45,925
No	79,018 (85.3)	7426 (8.1)	6002 (6.5)	121 (0.1)	92,567
Open Access Gold (OAG)					
Yes	21,158 (78.3)	3367 (12.5)	2370 (8.8)	113 (0.4)	27,008
No	100,215 (90)	6310 (5.6)	4856 (4.3)	103 (0.1)	111,484

In total, included publications were cited 1,825,365 times, with 92.5% of citations belonging to HIC-affiliated corresponding authors, and < 0.1% belonging to LIC-affiliated authors (*p* < 0.05). Of all articles that were published in orthopaedic journals indexed by SCIE, 89.3% were from HIC-affiliated authors and of those articles listed in ESCI-indexed journal, 74.4% were from HIC-affiliated authors. Compared to HIC-affiliated author publications, there were lower odds of articles being published in SCIE journals from authors affiliated with an UMIC (OR = 0.77; 95% CI: 0.72, 0.82), LMIC (OR = 0.17; 95% CI: 0.16, 0.18), and LIC institutions (OR = 0.14; 95% CI: 0.11, 0.19).

### Research funding

Authors of 45,925 (33.2%) articles acknowledged some funding source for the published study (Table 1). Compared to the HIC category, there were lower odds of funding noted for authors affiliated with an UMIC (OR = 0.57; 95% CI: 0.54, 0.60) and LMIC (OR = 0.38; 95% CI: 0.36, 0.40).

### Open access gold publication

Of all included articles, 19.5% were published as open access gold (OAG), of which 78.3% were HIC-affiliated in comparison to 12.5% UMIC, 8.8% LMIC, and 0.4% LIC (Table 1). Considering each income category, 17.4% of all HIC-affiliated articles had OAG in comparison to 34.8% of all UMIC, 32.8% of all LMIC, and 52.6% of LICs affiliated articles. Compared to HIC, there were increased odds of OAG publication for authors from UMICs (OR = 2.52; 95% CI: 2.41, 2.64), LMICs (OR = 2.32; 95% CI: 2.20, 2.44), and LICs (OR = 5.25; 95% CI: 4.01, 6.86) (*p* < 0.05).

## Discussion

This study aimed to investigate the quantity, quality, and trends of peer-reviewed publications in orthopaedic surgery based on country income stratification of each author’s institutional affiliation. Our findings align with a prior study reporting that only 0.1% of articles published in 76 orthopaedic surgery journals originated from LICs and 2.7% from LMICs [17]. Using a larger pool of journals for our study along with a more in-depth bibliometric analysis, we noted that a higher proportion of publications from lower income countries were in the “lower impact” journals, i.e., in ESCI versus SCIE [18]. To be included in the SCIE collection, journals must also be evaluated by four additional criteria (compared to ESCI journals) and yield a Journal Impact Factor (JIF) [10, 11]. Thus SCIE status journal perhaps has greater credibility amongst researchers and readers. In fact, some bibliometric studies in orthopedics only use journals listed in the SCIE collections [19, 20]. Publishing in SCIE journals with a high impact factor can have a positive downstream effect, especially those with Open Access, with an increased odd of future citations and further increase the visibility of the findings of the conducted research. Other contributors to lower research productivity amongst LIC-affiliated authors could be the excessive burden of care carried by limited number of orthopedic surgeons in the region, lack of appropriate resources and infrastructure to conduct research, poor follow-up visits due to limited access, long travel times with transportation barriers for patients and availability of alternative non-allopathic remedies offered by local non-traditional providers. Such factors can adversely impact the typical requirements for a high-quality evidence-based research manuscript that is sought by journal reviewers, editors and readers [21, 22].

Limitations of our study include the exclusion of non-English articles. Furthermore, while we evaluated all articles in WoS collections, including SCIE and ESCI collections, these may not include certain local or regional journals where some of these authors may also publish. One can also critique that the statistical methodology that we used for assessing articles with authors from multiple income-countries. An additional limitation of this study is inherent in the analysis and framing of our interpretation through the lens of our current understanding of barriers that exist for LIC/LMIC authors, as the majority of authors of the current study are also from a high-income country, although few of us have worked in resource-limited environments and actively participate in global orthopaedic education and training. Future research in this field should integrate perspectives from authors from lower income countries to further understand the barriers and propose solutions that can be better aligned with the burden of musculoskeletal disease globally.

Stratifying by income category, HIC-affiliated authors had the largest absolute increase in number of published articles (9107–14,619), compared to UMIC-affiliated (495–1214), LMIC-affiliated (406–874), and LIC-affiliated (4–28) authors. There could be several potential reasons for these findings. A number of journals currently charge processing fees for open access publication, which can be a barrier for scholars, especially in LICs or LMICs [23]. While waiving APC is a step in the right direction, articles written in “non-native like” English experience unfair scrutiny in the review process due to grammar or syntax that may prevent them from progressing past the submission phase [24]. Although the English language is widely spoken across the world, given the diversity of native languages across health care systems and their patients globally, leaders of scientific journals must acknowledge and work around the inherent barriers of assuming immaculate English writing proficiency in submissions. There are several supportive services that journals can and sometimes do provide for authors to mitigate these barriers. For example, journals can provide cost-free or subsidized services in proofreading or translating submitted manuscripts with authors from non-English speaking countries. Similarly, publishers can consider allowing authors to use artificial intelligence programs such as Chat GPT within guidelines (to avoid an unfair advantage) to aid non-English speaking authors to write select portions of the research manuscripts, provided that use of such a tool is acknowledged by the authors in the Materials and methods section and does not create an unfair advantage [25]. Regardless, the content and potential impact of the reported findings of submitted manuscripts, rather than their presentation alone should carry the greatest weight in the eyes of reviewers and editors. Journals can similarly make greater efforts to publicize such resources for potential authors from non-English speaking LICs or LMICs, such as available fee waivers and language editing support, including information brochures and e-information for attendees at local or regional conferences. The leadership of various journals can also implement protocols that reduce bias in the review process. Expanding the editorial board and reviewer pool to increase representation from surgeons of LICs/LMICs countries is another way to ensure a more equitable peer-review process. Additionally, including representatives from an LIC or LMICs in the reviewer panel for any submission from a LICs or LMICs can make the review process more fair and contextual [26].

Research studies conducted in lower income countries can be relevant for surgeons practicing in higher income countries. For instance, scholars from LICs and LMICs often identify cost-effective treatment options that are more accessible to their patients, modify follow-up and rehabilitation, and inform unique patient outcomes influenced by local culture and practices [21, 22, 27]. Surgeons practicing in high-resource countries can benefit from learning about musculoskeletal health in low-resource settings through the notion of “frugal innovations” and “transformative learning” as explained by Mezirow, which calls for learning experiences that challenge readers’ preconceived ideas, often by confronting them with “disorienting dilemmas” [28, 29]. A recent perspective paper suggests that to decolonize global health partnerships, all stakeholders need to critically reflect on “problematic frames of reference – sets of fixed assumptions and expectations” to foster both self-awareness and a deeper awareness of others [23]. In an era where health-care costs and medical waste are rapidly increasing, learning from LIC/LMIC partners and adopting frugal innovations is very relevant [22].

We also noted that of the 133,718 unique articles, only 74 articles had authors from both an HIC- and LIC-affiliated institutions, and 1762 had HIC- and LMIC-affiliated authors. Academic partnerships between low-resource and high-resource stakeholders can improve the research capacity by establishing healthy, mutually beneficial partnerships that focus on bidirectional exchange [30–32]. However, in the long-term, it is essential that scholars from LICs or LMICs build a sustainable and locally relevant infrastructure and acquire skills to independently ask critically important and answerable research questions, execute using sound methodology, and publish their findings in appropriate journals that have a broad readership base. Autonomy in conducting to publishing research is the most important goal to yield sustainable research output from scholars in LICs or LMICs in the long-term.

In conclusion, this study highlights the substantial and persistent disparities in orthopaedic surgery research based on the country income status of the authors’ affiliated institution. Efforts should be made to increase the representation and visibility of peer-reviewed articles authored by investigators from low-income countries in order to improve the musculoskeletal health of people worldwide.

## Appendix A

Syntax for WoS Expanded API or WoS Advanced Search:

SO=ACTA CHIRURGIAE ORTHOPAEDICAE ET TRAUMATOLOGIAE CECHOSLOVACA or SO=ACTA ORTHOPAEDICA or SO=ACTA ORTHOPAEDICA BELGICA or SO=ACTA ORTHOPAEDICA ET TRAUMATOLOGICA TURCICA or SO=acta ortopedica brasileira or SO=ADVANCES IN ORTHOPEDICS or SO=AMERICAN JOURNAL OF SPORTS MEDICINE or SO=ANNALS OF JOINT or SO=ARCHIVES OF BONE "AND" JOINT SURGERY ABJS or SO=ARCHIVES OF ORTHOPAEDIC "AND" TRAUMA SURGERY or SO=ARCHIVES OF OSTEOPOROSIS or SO=ARCHIVES OF TRAUMA RESEARCH or SO=ARTHROSCOPY TECHNIQUES or SO=ARTHROSCOPY THE JOURNAL OF ARTHROSCOPIC "AND" RELATED SURGERY or SO=ASIAN JOURNAL OF ENDOSCOPIC SURGERY or SO=ASIAN SPINE JOURNAL or SO=ASIA PACIFIC JOURNAL OF SPORT MEDICINE ARTHROSCOPY REHABILITATION "AND" TECHNOLOGY or SO=BMC MUSCULOSKELETAL DISORDERS or SO=BONE JOINT JOURNAL or SO=BONE JOINT RESEARCH or SO=BRAZILIAN JOURNAL OF PHYSICAL THERAPY or SO=BULLETIN OF THE HOSPITAL FOR JOINT DISEASES or SO=CARTILAGE or SO=CHINESE JOURNAL OF TRAUMATOLOGY or SO=CLINICAL BIOMECHANICS or SO=CLINICAL JOURNAL OF SPORT MEDICINE or SO=CLINICAL MEDICINE INSIGHTS ARTHRITIS "AND" MUSCULOSKELETAL DISORDERS or SO=CLINICAL ORTHOPAEDICS "AND" RELATED RESEARCH or SO=CLINICAL SPINE SURGERY or SO=CLINICS IN ORTHOPEDIC SURGERY or SO=CLINICS IN PODIATRIC MEDICINE "AND" SURGERY or SO=CONNECTIVE TISSUE RESEARCH or SO=CURRENT ORTHOPAEDIC PRACTICE or SO=CURRENT REVIEWS IN MUSCULOSKELETAL MEDICINE or SO=EFORT OPEN REVIEWS or SO=EUROPEAN CELLS MATERIALS or SO=EUROPEAN SPINE JOURNAL or SO=FOOT ANKLE INTERNATIONAL or SO=FOOT "AND" ANKLE CLINICS or SO=FOOT "AND" ANKLE SURGERY or SO=GAIT POSTURE or SO = GERIATRIC ORTHOPAEDIC SURGERY REHABILITATION or SO=GLOBAL SPINE JOURNAL or SO=HAND CLINICS or SO=HAND SURGERY REHABILITATION or SO=HIP INTERNATIONAL or SO=INDIAN JOURNAL OF ORTHOPAEDICS or SO=INJURY INTERNATIONAL JOURNAL OF THE CARE OF THE INJURED or SO=INTERNATIONAL JOURNAL OF PHYSIOTHERAPY or SO=INTERNATIONAL JOURNAL OF SURGERY OPEN or SO=INTERNATIONAL ORTHOPAEDICS or SO=ISOKINETICS "AND" EXERCISE SCIENCE or SO=JOINT DISEASES "AND" RELATED SURGERY or SO=JOR SPINE or SO=JOURNAL OF ARTHROPLASTY or SO=JOURNAL OF BACK "AND" MUSCULOSKELETAL REHABILITATION or SO=JOURNAL OF BONE "AND" JOINT SURGERY AMERICAN VOLUME or SO=JOURNAL OF CHILDRENS ORTHOPAEDICS or SO=JOURNAL OF FOOT ANKLE SURGERY or SO=JOURNAL OF FOOT "AND" ANKLE RESEARCH or SO=JOURNAL OF HAND SURGERY AMERICAN VOLUME or SO=JOURNAL OF HAND SURGERY EUROPEAN VOLUME or SO=JOURNAL OF HAND THERAPY or SO=JOURNAL OF HIP PRESERVATION SURGERY or SO=JOURNAL OF KNEE SURGERY or SO=JOURNAL OF ORTHOPAEDIC SPORTS PHYSICAL THERAPY or SO=JOURNAL OF ORTHOPAEDIC RESEARCH or SO=JOURNAL OF ORTHOPAEDIC SCIENCE or SO=JOURNAL OF ORTHOPAEDIC SURGERY or SO=JOURNAL OF ORTHOPAEDIC SURGERY "AND" RESEARCH or SO=JOURNAL OF ORTHOPAEDIC TRANSLATION or SO=JOURNAL OF ORTHOPAEDIC TRAUMA or SO=JOURNAL OF ORTHOPAEDICS or SO=JOURNAL OF ORTHOPAEDICS "AND" TRAUMATOLOGY or SO=JOURNAL OF ORTHOPAEDICS TRAUMA "AND" REHABILITATION or SO=JOURNAL OF OSTEOPOROSIS or SO=JOURNAL OF PEDIATRIC ORTHOPAEDICS or SO=JOURNAL OF PEDIATRIC ORTHOPAEDICS PART B or SO=JOURNAL OF PHYSIOTHERAPY or SO=JOURNAL OF PLASTIC SURGERY "AND" HAND SURGERY or SO=JOURNAL OF SHOULDER "AND" ELBOW SURGERY or SO=JOURNAL OF THE AMERICAN ACADEMY OF ORTHOPAEDIC SURGEONS or SO=JOURNAL OF THE AMERICAN ACADEMY OF ORTHOPAEDIC SURGEONS GLOBAL RESEARCH "AND" REVIEWS or SO=JOURNAL OF THE AMERICAN PODIATRIC MEDICAL ASSOCIATION or SO=JOURNAL OF WRIST SURGERY or SO=KNEE or SO=KNEE SURGERY SPORTS TRAUMATOLOGY ARTHROSCOPY or SO=MALAYSIAN ORTHOPAEDIC JOURNAL or SO=MINERVA ORTOPEDICA E TRAUMATOLOGICA or SO=MLTJ MUSCLES LIGAMENTS "AND" TENDONS JOURNAL or SO=OBERE EXTREMITAET SCHULTER ELLENBOGEN HAND UPPER EXTREMITY SHOULDER ELBOW HAND or SO=OPERATIVE ORTHOPADIE UND TRAUMATOLOGIE or SO=OPERATIVE TECHNIQUES IN ORTHOPAEDICS or SO=ORTHOPADE or SO=ORTHOPAEDIC JOURNAL OF SPORTS MEDICINE or SO=ORTHOPAEDIC NURSING or SO=ORTHOPAEDIC SURGERY or SO=ORTHOPAEDICS TRAUMATOLOGY SURGERY RESEARCH or SO=ORTHOPEDIC CLINICS OF NORTH AMERICA or SO=ORTHOPEDIC RESEARCH "AND" REVIEWS or SO=ORTHOPEDIC REVIEWS or SO=ORTHOPEDICS or SO=OSTEOARTHRITIS "AND" CARTILAGE or SO=OSTEOLOGIE or SO=PHYSICAL THERAPY or SO=PHYSICIAN "AND" SPORTSMEDICINE or SO=PROSTHETICS "AND" ORTHOTICS INTERNATIONAL or SO=SICOT J or SO=SKELETAL RADIOLOGY or SO=SPINE or SO=SPINE JOURNAL or SO=SPORTVERLETZUNG SPORTSCHADEN or SO=STRATEGIES IN TRAUMA "AND" LIMB RECONSTRUCTION or SO=TECHNIQUES IN FOOT "AND" ANKLE SURGERY or SO=TECHNIQUES IN ORTHOPAEDICS or SO=TECHNIQUES IN SHOULDER "AND" ELBOW SURGERY or SO=TRAVMATOLOGIYA I ORTOPEDIYA ROSSII or SO=WORLD JOURNAL OF ORTHOPEDICS or SO=ZEITSCHRIFT FUR ORTHOPADIE UND UNFALLCHIRURGIE.
