# Erratum to: “Peer-reviewed publications in orthopaedic surgery from lower income countries: A comparative analysis”

**DOI:** 10.1051/sicotj/2024014

**Published:** 2024-05-16

**Authors:** Sanjeev Sabharwal, Andrea Leung, Joel Johansen Bwemelo, Patricia Rodarte, Annelise S. Taylor, Gurbinder Singh, Josephine Tan, Richard Trott

**Affiliations:** 1 UCSF Benioff Children’s Hospital Oakland, Department of Orthopaedic Surgery 747 52nd Street Oakland CA 94609 USA; 2 Institute of Global Orthopaedics and Traumatology (IGOT) 2540 23rd Street, Building 7 San Francisco CA 94110 USA; 3 Muhimbili Orthopaedic Institute Kalenga Street West Upanga Dar es Salaam Tanzania; 4 University of California, San Francisco Library 530 Parnassus Ave San Francisco CA 94143 USA

Regarding this article, published on February 1, 2024, an error occurred in the authors affiliations:

The correct affiliation for the authors Annelise S. Taylor, Josephine Tan and Richard Trott is:

University of California, San Francisco Library, 530 Parnassus Ave, San Francisco, CA 94143, USA

The Publisher will ensure that all modifications will be correctly transmitted to the indexation bases.

The Publisher apologizes for this mistake.

